# Proliferating cell nuclear antigen (PCNA), p53 and MDM2 expression in Hodgkin’s disease

**DOI:** 10.1590/S1516-31802007000200003

**Published:** 2007-03-04

**Authors:** Gevina Silva Pinheiro, Maria Regina Régis Silva, Celso Arrais Rodrigues, José Kerbauy, José Salvador Rodrigues de Oliveira

**Keywords:** Proliferating cell nuclear antigen, p53 genes, Proto-oncogene proteins c-mdm2, Hodgkin disease, Immunohistochemistry, Antígeno nuclear de célula em proliferação, Genes p53, Proteínas proto-oncogênicas c-mdm2, Doença de Hodgkin, Imunohistoquímica

## Abstract

**CONTEXT AND OBJECTIVE:**

Tumor cells in Hodgkin’s disease (HD) express cell proliferation markers that are evaluated according to the oncogenes involved or the expression of their proteins. Correlations between the protein expression grade and clinical data are now important for disease prognosis.

**DESIGN AND SETTING:**

This was a retrospective analysis on proliferating cell nuclear antigen (PCNA), p53 and MDM2 (murine double minute-2) expression using immunohistochemistry, on formalin-fixed, paraffin-embedded tissues from diagnostic biopsies on 51 patients with HD. The study was conducted at the Division of Hematology and Transfusion Medicine, Hospital São Paulo, Universidade Federal de São Paulo.

**METHODS:**

Antigen expression was evaluated as the proportions of positive Hodgkin and Reed-Sternberg (HRS) cells and reactive lymphocytes (L), which were compared using Spearman correlation coefficients. The Friedman test was used for comparisons between the markers. The Pearson test was used to investigate associations between marker expression and clinical and laboratory parameters, marrow involvement, complete remission (CR) and overall survival (OS) rates.

**RESULTS:**

There was overexpression of antigen proteins in HRS, in relation to L (p < 0.001). In HRS, MDM2 was higher than p53 and PCNA (p < 0.003), while the latter two were equivalent. In L, p53 was lower than MDM2 and PCNA (p < 0.001), while the latter two were equivalent. There was no relationship between protein expression and clinical and laboratory variables or outcome.

**CONCLUSIONS:**

PCNA, p53 and MDM2 are tumor markers for HD, but showed no clinical or prognostic significance in our analysis.

## RESUMO

**CONTEXTO E OBJETIVO:**

As células tumorais da doença de Hodgkin (HD) são positivas para marcadores de proliferação celular que são analisados por seus genes e respectivas proteínas. A correlação entre a expressão destas proteínas e os parâmetros clínico-laboratoriais são, no momento, de importância para o prognóstico da doença.

**TIPO DE ESTUDO E LOCAL:**

Estudo retrospectivo da expressão do antígeno de proliferação celular (PCNA) e da p53 e MDM2 em tecidos obtidos ao diagnóstico, fixados por formol, embebidos em parafina de 51 pacientes com HD. O trabalho foi realizado na Divisão de Hematologia e Transfusão, Hospital São Paulo, Universidade Federal de São Paulo.

**MÉTODOS:**

As expressões antigênicas foram analisadas através da proporção de células de Hodgkin e células de Reed Sternberg (HRS) e linfócitos reacionais (L) positivos. A intensidade de expressão de cada proteína foi comparada entre L e HRS através do coeficiente de Spearman. A comparação da PCNA, p53 e MDM2 em L e HRS se fez pelo teste de Fiedman. As correlações entre variáveis clínico-laboratoriais, comprometimento da medula óssea, taxas de sobrevida geral e remissão clínica com as proteínas em HRS se fizeram pelo coeficiente de Pearson.

**RESULTADOS:**

Houve superexpressão das três proteínas em células HRS comparadas aos L (p < 0,001). Nas células HRS, a MDM2 foi maior que a p53 e a PCNA (p < 0,003), que foram equivalentes. Nos L, a p53 foi menor que a MDM2 e a PCNA (p < 0,001), que foram equivalentes Não houve relação entre as expressões das proteínas com as variáveis clínico-laboratoriais e sobrevida.

**CONCLUSÕES:**

PCNA, p53 e MDM2 são marcadores tumorais na HD, porém não mostraram significado clínico-prognóstico em nossa análise.

## INTRODUCTION

Hodgkin’s disease (HD) is a neoplasm that has the characteristic of containing a small number of scattered large multinucleated or mononucleated cells, designated Reed-Sternberg cells and Hodgkin cells respectively, residing in a heterogeneous admixture of inflammatory and accessory cells.^1,2^ The paucity of Hodgkin and Reed-Sternberg (HRS) tumor cells initially made it difficult to determine the origins of these cells.^1-3^ However, high-density genomic expression and immunoglobulin H (IgH) variable-region gene rearrangements using single-cell analysis obtained by microdissection techniques have now demonstrated an origin in germinative B cell centers.^4-9^

There are few previous studies in the literature evaluating cell proliferation and this may be explained by the complex nature of this neoplasm and its heterogeneous cell com-position.^10^ It has already been described that, in HD, HRS cells express proliferating cell nuclear antigen (PCNA) and p53 in more than 50% of the cases and that these expressions may play a role in the pathogenesis of the disease.^11^ PCNA is a cell cycle-associated protein that interferes with cell proliferation in normal and tumor cells. It is an essential protein in DNA repair. PCNA is detected by positive reaction for the monoclonal antibody PC-10.^10,11^ PCNA activity in DNA repair increases resistance to chemotherapy in which the cytotoxicity depends on its integrity.^12^ PCNA expression in relapsed HD is greater than at diagnosis.^12^

Wild-type p53 protein encoded by the *p53* gene acts in the cell cycle to interrupt it at the G1 phase.^13^ This suppressive activity allows DNA repair in injured cells and avoids apoptosis.^14^ Deletion or mutation of *p53* is classically associated with p53 tumor activity.^13-15^ Mutated p53 has a mean half-life of six to eight hours and no suppressive action, and it is easily detectable by immunohistochemistry methods. Mutated p53 replaces the wild-type p53 that is present in low intracellular concentrations, and has a shorter half-life of 20 min.^13-16^ *p53* mutation is missense and occurs mainly between exons 5 and 9.^15,16^ *p53* mutations lead to loss of suppressive function, thereby accelerating tumor genesis.^13,14^ p53 overexpression has been widely detected in HD, even in the absence of known *p53* mutations.^12,15,17-20^ The MDM2 (murine cell double minute-2) gene encodes the p90 protein, which binds to wild-type p53^17,18^ to inhibit its suppressive activity in transcription.^19,20^ Associated expression of p53 and MDM2 is present in more than 90% of HD cases.^17-19,21^

The Epstein-Barr virus (EBV) genome is found in up to 50% of cases of HD.^22^ EBNA-1 (Epstein-Barr nuclear antigen-1) bonds with p53 and it has been suggested that EBNA-1 expression results from that interaction.^23^ The exact role of EBV in HD genesis is not clear yet.^22,23^

## OBJECTIVE

The present study had the aims of evaluating p53, PCNA and MDM2 protein expression using immunohistochemical methods, on formalin-fixed, paraffin-embedded preserved tissue samples used for HD diagnosis, and correlating these expressions with clinical and laboratory parameters in order to evaluate their impact on HD outcome.

## PATIENTS AND METHODS

### Patients

Our sample consisted of 126 patients with HD that were followed up over the period from Janaury 1992 to December 1996 at Hematology Division of Unifesp/EPM. The admissions for the first-line treatment of these patients were from December 1976 to December 1996. Sufficient paraffin-embedded blocks of diagnostic tissues were available in relation to 51 patients from this sample for performing new histological analyses with hematoxylin-eosin staining and for making immunohistochemistry slides for PCNA, p53 and MDM2 analysis. Fifty-one other patients out of the remaining 75 were randomly selected as a control group. The paraffin-embedded blocks were obtained from the Pathology Department of Unifesp.

The patients’ records were retrospectively reviewed to collect data on gender, age, Ann Arbor clinical stage, B symptoms (fever, weight loss and night sweating), histological subtype, hemoglobin level, white blood count (WBC), erythrocyte sedimentation rate (ESR), alkaline phosphates and bone marrow involvement. First complete remission (CR) and overall survival (OS) were also evaluated (Table 1). Nine of these patients (six in the study group and three in the control group) were not evaluated with regard to the achievement of first CR and OS, but they had diagnostic and staging data available and therefore they were included because of these criteria.

**Table 1:** Dichotomization and coding of variables evaluated

	Code	
Variable	0	1
Gender	Male	Female
Age	< 40 years	≥ 40 years
Histological subtype	LP and NE	MC and LD
Clinical stage	Ι and ΙΙ	ΙΙΙ and ΙV
B Symptoms	No	Yes
Hemoglobin Level	< 12 g	≥ 12 g
WBC	< 10X10^9^/l	≥ 10 X10^9^/l
ESR	< 20 mm	≥ 20 mm
Alkaline phosphatase	< 150 U	≥ 150 U
Bone marrow involvement*	Negative	Positive
%HRS + MDM2	< median (= 59.37%)	≥ median
%HRS + PCNA	< median (= 52.72%)	≥ median
%HRS + p53	< median (= 53.45%)	≥ median

LP = lymphocyte predominance; NE = nodular sclerosis; MC = mixed cellularity; LD = lymphocyte depletion; WBC = whole blood count; ESR = erythrocyte sedimentation rate; HRS = Hodgkin and Reed-Sternberg cells; MDM2 = murine double minute-2 antigen; PCNA = proliferating cell nuclear antigen

* bone marrow infiltration: positive: presence of bone marrow involvement of HD; negative: absence of bone marrow HD infiltration.

The first patient was treated and followed up starting in 1976 and the remainder from 1980 onwards. Subsequently, the treatment protocols changed over the course of time. Patients treated before 1985 received chemotherapy using the MOPP (mechlorethamine, vincristine, prednisone and procarbazine) protocol, while those treated after that year received either the MOPP/ABV (MOPP plus doxorubicin, bleomycin and vincristine) hybrid protocol or the MOPP/ABVD (ABV plus dacarbazine) alternative regimen.^24^ Involved-field radiation therapy was performed on all stage I and II patients and on most stage III patients, except those with bulky disease, who received extended-field radiation therapy.^24^

There were no significant differences between the control and study groups with regard to the following variables: age (p = 0.19), hemoglobin level (p = 0.99), WBC (p = 0.53), ESR (p = 0.37) A and B symptoms (p = 0.14), bone marrow infiltration (p = 0.46), likelihood of achieving the first complete remission (CR) (p = 0.68), overall survival (OS) (p = 0.83) and stages (I + II, III + IV) (p = 0.99). In relation to histological subtypes (LP + NE), (MC + LD) (LP = lymphocyte predominance; NE = nodular sclerosis; MC = mixed cel-lularity; LD = lymphocyte depletion) (p = 0.03), greater frequency of NE in the study group than in the control group was observed.

Forty-five out of 51 patients in the study group and 48 out of 51 in the control group could be evaluated regarding achievement of first CR and OS. There were 12 deaths: 7/48 (14%) and 5/45 (11%) in the control and studied groups, respectively.

This study was approved by the Ethics Committee of Universidade Federal de São Paulo, and informed consent was obtained from all subjects who were still alive.

### Methods

All the lymph node biopsies were preserved in formalin and embedded in paraffin. Samples were then restained using the hematoxylin-eosin method and reviewed by two hematopathologists. The minimum immunohistochemistry panel consisted of anti-CD30 and CD15 antibodies. Once HD had been confirmed, PCNA, p53 and MDM2 antibodies were also included. The tissue sections underwent routine treatment. Antigenic recovery was performed by immersing the slides in 0.001 M citrate buffer (pH 6.0) and heating in a microwave oven at maximum power for 45 minutes. After cooling for 20 minutes at room temperature, the slides were washed under running water for five minutes and distilled water for a further five minutes. Endogenous peroxidase was then blocked by immersing the slides in a 0.006% hydrogen peroxide solution for two nine-minute periods, each followed by rinsing using distilled water and phosphate buffer solution (PBS).

Monoclonal antibodies were added at the concentrations recommended by the manufacturer, as follows. For PCNA: PC-10 (Dako, catalog no. MO-879) at 1/200 concentration for 18 hours at 4° C, followed by PBS washing; for MDM2: MDM2 (Novocastra, catalog no. NCR-MDM2) at 1/200 concentration; and for p53: DO7 (Novocastra, catalog no. NCL-p53DO7) and BP (Novocastra, catalog no. NCL-p53BP), at 1/50 concentration for both, incubated for 18 hours at 4º C. The secondary antibody was biotinylated rabbit anti-mouse (Dako, catalog no. E-0354), at 1/100 concentration. Development occurred after incubation with the streptavidin-biotin-peroxidase-ABC complex (Dako, catalog no. K-377 A-B), at 1/200 concentration for 30 minutes at 37° C, followed by development on a chromogenic substrate of 3,3’ diaminobenzidine (PAB-Sigma, catalog no. D-5637) at 0.006% concentration in PBS, adding 100 µl of hydrogen peroxide at 30 volumes for each 10 ml of solution.^25^

The PCNA samples were counterstained with Fast-Green (Inlab, catalog no. 3870) and p53 and MDM2 with Harris hematoxylin. Negative controls were obtained from distinctive parts of the same slide, by omission of the tested monoclonal antibody. Positive controls were obtained from breast cancer slides that were known to be positive for p53, MDM2 and PCNA, which were provided by the pathology department of our institution. The number of positive cells was determined from the HRS and L counts. Positive cells were defined as all cells with any pattern of nuclear staining; negative cells were those without this. Two of the present authors performed the counting, using an optical microscope at a magnification of 1000 times, with a 100-dot integrator (Zeiss), in five randomly chosen different fields. In every case, a positive-negative ratio was calculated for HRS and L after specific and general proportion ratios for positive and negative L and HRS cells had been obtained.

### Statistical analysis

The tests used were the Wilcoxon test and Spearman coefficient to compare marker expression between HRS and L, Friedman’s test to determine differences between the markers (PCNA, p53 and MDM2) in HRS and L, and Pearson’s chi-squared test to evaluate clinical and laboratory variables. CR achievement and OS were calculated by the Kaplan-Meier method and the curves were compared using log-rank tests. Multivariate analysis was based on the Cox regression model. Variables were dichotomized for univariate and multivariate analysis regarding CR achievement and OS. Two-tailed p < 0.05 was considered statistically significant.

## RESULTS

There was preponderance of male subjects: 34 patients were male (67%) and 17 were female (33%). Their ages ranged from 9 to 88 years (median: 32 years). Nodular sclerosis was the most common histological subtype and was found in 38 patients (74.5%), followed by mixed cellularity in seven patients (13.7%), lymphocyte depletion in three (5.8%) and lymphocyte predominance in three (5.8%). Ann Arbor clinical stage I was found in three patients (5.8%), II in 14 (27.4%), III in 16 (31.3%) and IV in 18 (35.3%). Therefore, 66.6% had advanced disease (stage III or IV) at diagnosis. Moreover, 36 out of the 51 patients (72.5%) presented with B symptoms.

The first-line therapy was thus the MOPP/ ABV hybrid for 39 patients, MOPP for six patients and the MOPP/ABVD alternative scheme in four cases.^24^ Involved-field radiation therapy was performed in 28 cases and extend-ed-field radiation therapy in 12 patients.

The median hemoglobin level was 11.7 g/dl (ranging from 4.4 g/dl to 16.3 g/dl), median WBC was 8 × 10^9^/µl, median ESR was 58 mm/h and median alkaline phosphatase was 233 U/l. Bone marrow analysis was negative in 40 patients, positive in eight and not available in three patients.

**Graph 1:**
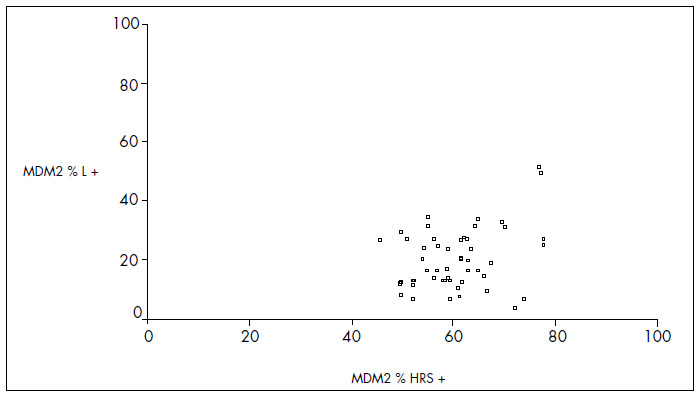
Dispersion of proportional values for MDM2 expression between Hodgkin and Reed-Sternberg (HRS) cells and lymphocytes (L).

**Graph 2:**
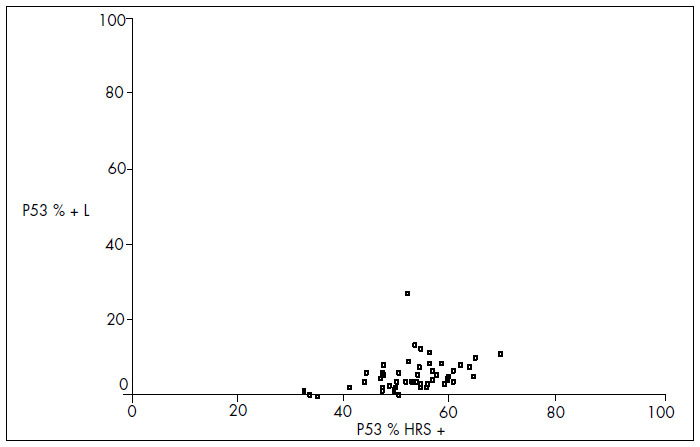
Dispersion of proportional values for p53 expression between Hodgkin and Reed-Sternberg (HRS) cells and lymphocytes (L).

**Graph 3:**
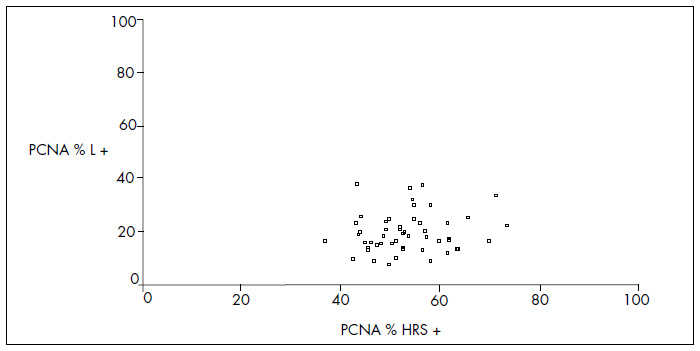
Dispersion of proportional values for proliferating cell nuclear antigen (PCNA) expression between Hodgkin and Reed-Sternberg (HRS) cells and lymphocytes (L).

**Figure 1:**
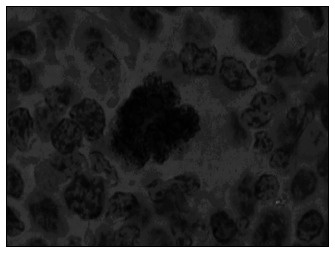
Photomicrograph of lymph node showing nuclear p53 expression in Reed-Sternberg cells, developed by peroxidase; 1000x magnification.

**Figure 2:**
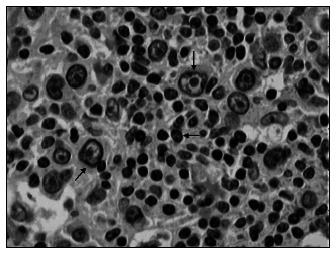
Photomicrograph of lymph node showing nuclear MDM2 expression in Hodgkin cells (upper right and left arrows) and lymphocytes (lower right arrow), developed by peroxidase; 400x magnification.

**Figure 3:**
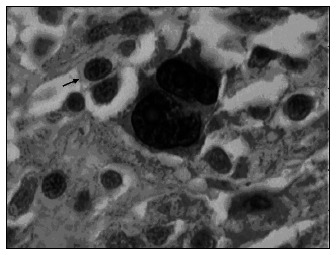
Photomicrograph showing nuclear PCNA expression in Reed-Sternberg cells and some Hodgkin cells (arrow), developed by peroxidase; 1000x magnification.

**Graph 4:**
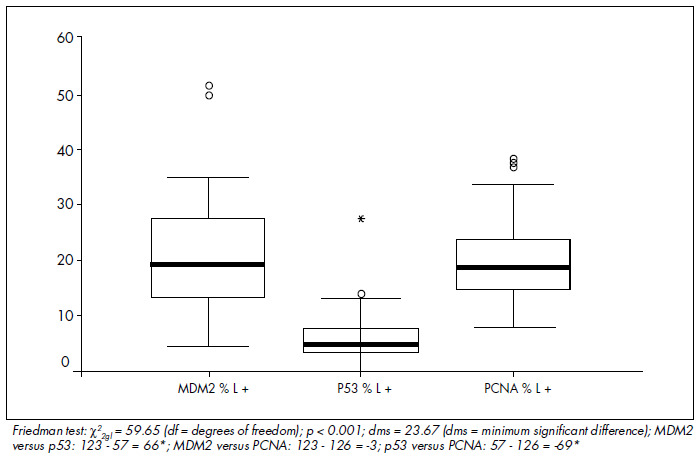
Comparison of positive lymphocyte (L) expression for MDM2, p53 and proliferating cell nuclear antigen (PCNA).

The mean MDM2 expression was 60% in HRS and 20.7% in L (p < 0.001). For p53, the mean expression was 52.9% in HRS and 5.9% in L (p < 0.001). For PCNA, the mean expression was 53.4% in HRS and 20% in L (p < 0.001) (Graphs 1, 2 and 3 and Tables 1, 2, 3 and 4). The expression of these tumor markers in HRS was predominantly in the nucleus, and was more positive in tumors than in reactive lymphocytes, for all three markers (Figures 1, 2 and 3 and Graphs 1, 2, 3 and 4).

**Table 2:** Univariate analysis. Odds ratios (OR) for the percentage of positivity in Hodgkin and Reed-Sternberg cells (%HRS+) (≥ mean or < mean) for PCNA

Variable	N	OR	95% CI	p
%HRS+ for MDM2	51	0.35	0.113–1.095	0.071
%HRS+ for p53	51	2.40	0.780–7.389	0.127
Alkaline phosphatase	46	2.31	0.632–8.469	0.205
WBC	51	1.56	0.496–4.898	0.448
ESR	42	0.51	0.076–3.409	0.486
Histological subtype	51	1.58	0.386–6.423	0.526
Age	51	0.73	0.171–3.093	0.666
Clinical stage	51	1.27	0.394–4.063	0.692
Hemoglobin level	51	1.10	0.360–3.358	0.867
Gender	51	0.94	0.304–2.886	0.910
B symptoms	51	1.05	0.232–4.739	0.952
Bone marrow involvement	48	1.00	0.219–4.564	> 0.999

OR = odds ratio; CI = confidence interval; HRS = Hodgkin and Reed-Sternberg cells; PCNA = proliferating cell nuclear antigen, WBC = whole blood count; ESR = erythrocyte sedimentation rate.

**Table 3:** Univariate analysis. Odds ratios (OR) for the percentage of positivity in Hodgkin and Reed-Sternberg cells (%HRS+) (≥ mean or < mean) for MDM2

Variable	n	OR	95% CI	^p^
%HRS+ for PCNA	51	0.35	0.113–1.095	0.071
Alkaline phosphatase	46	0.31	0.080–1.204	0.091
Clinical stage	51	1.81	0.556–5.886	0.324
B symptoms	51	0.57	0.121–2.702	0.481
Histological subtype	51	1.58	0.386–6.423	0.526
Age	51	0.73	0.171–3.093	0.666
Hemoglobin level	51	0.80	0.260–2.431	0.688
WBC	51	0.79	0.255–2.476	0.691
ESR	42	0.78	0.117–5.257	0.802
%HRS+ for p53	51	0.92	0.308–2.769	0.886
Bone marrow involvement	48	0.90	0.198–4.131	0.897
Gender	51	0.94	0.304–2.886	0.910

OR = odds ratio; HRS = Hodgkin and Reed-Sternberg cells; PCNA = proliferating cell nuclear antigen; CI = confidence interval; WBC = whole blood count; ESR = erythrocyte sedimentation rate.

**Table 4:** Univariate analysis. Odds ratios (OR) for the percentage of positivity in Hodgkin and Reed-Sternberg cells (%HRS+) (≥ mean or < mean) for p53

Variable	n	OR	95% IC	p
%HRS+ for PCNA	51	2.40	0.780–7.389	0.127
Histological subtypes	51	0.34	0.076–1.483	0.150
Age	51	0.41	0.091–1.876	0.252
Clinical stage	51	0.62	0.192–2.020	0.324
B symptoms	51	1.92	0.406–9.046	0.411
Bone marrow involvement	48	0.54	0.114–2.584	0.443
WBC	51	1.56	0.496–4.898	0.448
Hemoglobin level	51	1.52	0.496–4.685	0.462
ESR	42	1.42	0.212–9.518	0.717
Alkaline phosphatase	46	1.13	0.322–3.983	0.845
%HRS+ for MDM2	51	0.92	0.308–2.769	0.886
Gender	51	0.94	0.304–2.886	0.910

OR = odds ratio; HRS = Hodgkin and Reed-Sternberg cells; CI = confidence interval; PCNA = proliferating cell nuclear antigen; WBC = whole blood count; ESR = erythrocyte sedimentation rate; MDM2 = murine double minute-2 antigen.

**Graph 5:**
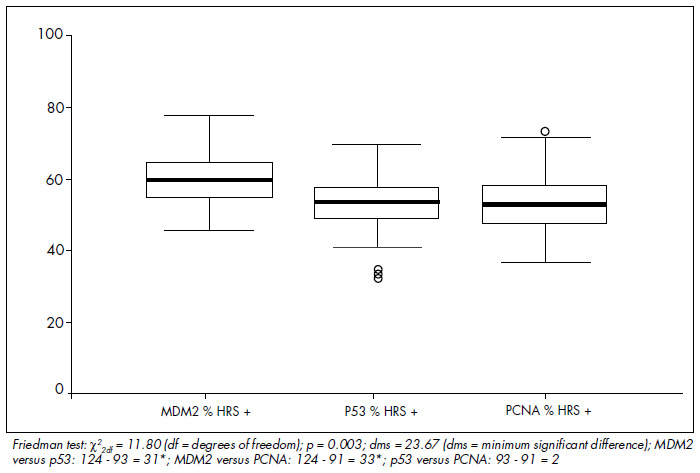
Comparison of positive Hodgkin and Reed-Sternberg (HRS) cell expression for MDM2, p53 and proliferating cell nuclear antigen (PCNA).

PCNA, p53 and MDM2 were not associated with gender, age, clinical stage, B symptoms, histological subtype, hemoglobin level, WBC, alkaline phosphatase, ESR or bone marrow involvement (Tables 2, 3 and 4). Univariate analysis between the proportions of marker expression in the tumor cells and all other variables investigated showed tendencies to correlate between MDM2 and PCNA (p = 0.07) (Graph 5, Table 2); PCNA and p53 (p = 0.12) (Graph 5 and Tables 3 and 4); alkaline phosphatase index higher than 150 U and MDM2 (p = 0.09); and unfavorable histology and p53 (p = 0.15) (Tables 1, 2, 3 and 4), but there was no association of marker expression between MDM2 and p53 (p = 0.88 in the Kaplan-Meier test) (Graph 5 and Tables 3 and 4). Multivariate statistical analysis also showed no significant differences.

The expression of different markers had no significant influence on CR achievement: p53 (p = 0.49); MDM2 (p = 0.59) and PCNA (p = 0.62). No significant influence on OS was found in relation to PCNA (p = 0.13), MDM2 (p = 0.21) or p53 (p = 0.54). The median OS for the study and control groups has not yet been reached. Therefore, we used the whole available population of 93 patients to analyze any possible associations for first CR and OS in relation to the other variables. The univariate analysis for the likelihood of achieving the first CR showed that B symptoms (p < 0.001), WBC ≥ 10 × 10^9^/µl (p = 0.01), stage (III + IV) (p = 0.01) and hemoglobin < 12 g/dl (p = 0.04) negatively influenced the possibility of achieving this. The results for bone marrow infiltration (p = 0.07) and age (p = 0.17) were close to reaching significance. The Cox regression model indicated that B symptoms (p = 0.001) and WBC ≥ 10 × 10^9^/µl (p = 0.01) were independent from the other analyzed variables in relation to the first CR.

All the patients without B symptoms (n = 22) and those with ESR less than 20 (n = 7) were still alive at the end of this study. However, these findings made it impossible to input these variables for multivariate analyses for OS.

In the univariate analysis, marrow involvement (p = 0.05), hemoglobin < 12 g/dl (p = 0.06), WBC ≥ 10 × 10^9^/µl (p = 0.07) and male sex (p = 0.1) showed tendencies towards lower OS. The log rank test demonstrated that bone marrow infiltration (p = 0.002), B symptoms (p = 0.03) and hemoglobin < 12 g/dl (p = 0.04) had a negative influence on OS. With regard to the CR ratios, 33/45 (73.3%) in the study group and 36/48 (75%) among the controls achieved CR; 6/45 (13.3%) in the study group and 3/48 (6.2%) among the controls obtained partial response; and 6/45 (13.3%) in the study group and 9/48 (18.7%) among the controls failed to achieve remission after the first treatment measures.

## DISCUSSION

HD is a unique human neoplasm consisting of a benign component of lymphocytes, plasmacytes, eosinophils and neutrophils with evident cellular polymorphism, in association with a much smaller component of tumor cells (HRS) comprising around 1% of the tumor mass.^1-3^

The cell proliferation rate is an important parameter for better understanding of the clinical picture and for guiding the appropriate therapy. PCNA monoclonal antibodies allow cell proliferation to be evaluated in clinical practice using immunohistochemical methods.^10-12^ In non-neoplastic small lymphocytes in HD tissues, PCNA expression is always low.^10-12,26,27^ Schmid et al.^28^ evaluated 23 cases of HD by double labeling with PC-10 monoclonal antibody and CD20 (B cells), and PC-10 and CD45RO (T cells), and found positivity in 50.4% of HRS and 4.3% of lymphocytes. HRS cells express PCNA in 5 to 100% of the cases. It has been demonstrated that the intensity of PCNA expression has an influence over clinical stages and response to treatment.^12,27,28^

We found PCNA-positive rates for HRS cells that ranged from 36.9% to 73.68% (median of 52.7%), which were similar to previously reported studies.^10-12^ We found higher values for PCNA in reactive lymphocytes than did most previous researchers (median of 18.8%).^12,27,28^ These differences are probably due to variations in the methods and discrepancies in manual cell counts between the individuals doing the counting.

There are many studies evaluating p53 and MDM2 expression in neoplasms. *p53* mutations without differences in protein expression have been reported.^15,17-19,21,22^ We characterized p53 using two different clones: BP and DO7. p53 expression was similar for these two clones (data not shown). This indicates that p53 expression in the wild type or in the mutated form may be evaluated using immunohistochemical methods either in fresh tissue or in formalin-fixed, paraffin-embedded preserved material. The application of this analysis depends on the characteristics and specificity of the monoclonal or polyclonal antibodies for p53.^15,16,20,26,29^

Latent EBV infection was initially suggested as a possible cause for *p53* mutation. This hypothesis has never been confirmed in clinical studies. The possibility of an association between latent EBV infection and p53 was first evaluated by Neidobitek et al.^30^ in 37 patients in 1993. Only seven cases that were positive for p53 were also positive for EBV proteins (EBER-1 or EBER-2). It is assumed that an association between p53 and EBV could be a consequence of EBV encoding proteins that are bonded to p53. Chilosi et al.^17^ investigated nuclear EBV using *in situ* hybridization for EBER-1 and immunohistochemistry for LMP-1 after double staining for p53 and MDM2. They found that 12 out of 72 patients who were positive for EBV were also positive for MDM2 and p53 concomitantly.

The role of MDM2 protein expression in activating/inhibiting the *p53* gene and in stabilizing p53 has also already been studied. Chen et al.^20^ showed that there was no relationship between MDM2 expression and *p53* mutations. MDM2 and p53 expression in HRS cells ranged from 30to 80%.^12,17,21,26^ In our study, concomitancy of p53 and MDM2 was found in only 37% of the cases. The lowest marker expression in reactive lymphocytes that we found, especially for p53, was comparable to previous descriptions.^12,16,17,21,26^ Positive lymphocyte reactions for PCNA and MDM2 at similar frequencies have also often been reported.^10-12,17,18,26,31^ Our positive results for PCNA, p53 and MDM2 in HRS cells are similar to those described by Martinez-Delgado et al.^29^ and Sánchez-Beato et al.^26^ In both of those studies, double staining for p53/MDM2 was performed and variable marker expression in HRS cells was also found.

Using histomorphometric techniques taking median values as thresholds, we found that the numerical expression of MDM2 was higher than those of PCNA and p53 in HRS cells, while there was no difference between PCNA and p53 expressions. The possible explanations for this finding are: 1) the antigen recovery technique utilized may have led to higher expression of epitopes for MDM2-specific antibodies; 2) MDM2 protein stability may be higher in formalin-fixed, paraffin-em-bedded tissue; 3) the characteristics of the antibodies utilized; and 4) the balance between the p53 and MDM2 pathways may favor MDM2 expression because of other mechanisms for p53 inhibition. We found a statistically significant lower proportion of lymphocytes were positive for p53 than were positive for PCNA and MDM2. This was also observed by Smolewski et al. in 1998,^31^ who described mean reactivity of lymphocytes for PCNA of 39.6%. According to most published studies, PCNA was expressed at higher frequencies than was p53.^21,26,31^ Histomorphometry was comparable to computed readings in the study by Sánchez-Beato et al.^26^ in 1996, for both markers.

Our data show that, in the "benign" inflammatory component of HD, there are proliferating and differentiating lymphocytes that are morphologically normal but carry positivity for proliferation markers near to HRS cells. These lymphocytes may correspond to precursors of HRS, as proposed by Hell et al.^27^ in 1993. The finding of higher expression of MDM2 than of PCNA and p53 in HRS cells may raise the possibility of a feedback mechanism between the *MDM2* and *p53* genes.^31-33^

The occurrence of spontaneous apoptosis, as evaluated by the TUNEL (*in situ* nick-end labeling) technique, ranged from 10 to 60% in HRS cells. The apoptosis index did not correlate with the histological and clinical findings, although a negative association with outcome was reported in 110 cases by Smolewski et al.^32^ Its presence is not influenced by the EBV genome in HRS cells. PCNA, p53, p21 and caspase 3 are associated with greater apoptosis indices, while BCL-2, MDM2, Rb-1 and P27 are not.^32-35^ In a smaller group of patients, it was confirmed that strong expression of PCNA, p53 and BCL-2 is associated with shorter OS and worse response to treatment.^31,33^ Brink et al.^35^ showed that the BCL-2+/p53– immunophenotype presented the worst prognosis, while the BCL-2–/p53– immunophenotype had five-year OS of more than 90% and the p53+/BCL-2+ immunophenotype had the best prognosis.^35^ High expressions of LMP1-EBV in HRS cells presented better response to therapy and better OS, while Rb-1 had worse prognosis. p53, BCL-2 and CD15 did not influence the outcome.^30,36-38^ We emphasize that MDM2 and PCNA, MDM2 and p53 expression, and also p53 expression versus unfavorable histology were close to reaching significance in the univariate analyses. These data show that, if a larger sample were used, a significant result might be found, as observed by other authors.^17-19,33-35,39^

B symptoms and WBC counts higher than 10x10^9^/µl were unfavorable independent factors for achieving the first CR. Bone marrow tumor infiltration, B symptoms and hemoglobin levels less than 12 g/dl had a negative influence on OS. These data have also been reported as negative prognostic factors for survival in HD.^24,32,34,39^ The present study was a retrospective analysis, but these findings have led us to attempt to be more specific regarding prognostic factors in this disease.^38^ The treatment for HD changed over the admission period for these patients, but the ratios of first CR and OS remained almost the same, because of the high sensitivity of the tumor to chemotherapy.^34^

## CONCLUSIONS

The methods we have used showed that p53, PCNA and MDM2 are expressed in HRS cells at different intensities. Further studies with larger series are needed to elucidate the influence of Epstein-Barr virus infection, frequency of Rb-1 gene loss and high expression of P21 on the clinical setting and the relationship of these three factors to treatment response in HD patients. The finding of no clear association between the antigen expression evaluated and the clinical data may be explained by the limited number of patients, the retrospective nature of the study and the high expression of these proteins, which made stratification and the result interpretation methods used more difficult.
